# Receipt of social services intervention in childhood, educational attainment and emergency hospital admissions: longitudinal analyses of national administrative health, social care, and education data in Wales, UK

**DOI:** 10.1186/s12889-024-20204-6

**Published:** 2024-10-21

**Authors:** Emily Lowthian, Graham Moore, Annette Evans, Rebecca Anthony, Muhammad Azizur Rahman, Rhian Daniel, Sinead Brophy, Jonathan Scourfield, Chris Taylor, Shantini Paranjothy, Sara Long

**Affiliations:** 1 Department of Education and Childhood Studies, School of Social Sciences, Swansea University, Swansea, Wales SA2 8PP UK; 2 Centre for Development, Evaluation, Complexity and Implementation in Public Health Improvement (DECIPHer), School of Social Sciences, SPARK, Cardiff University, Cardiff, CF24 4HQ UK; 3 Division of Population Medicine, Cardiff University, Cardiff, Wales CF14 4XN UK; 4 Wolfson Centre for Young People’s Mental Health, Cardiff University, Hadyn Ellis Building, Maindy Road, Cardiff, CF24 4HQ UK; 5 Research and Evaluation Division, Public Health Wales, 2 Capital Quarter, Tyndall Street, Cardiff, CF10 4BZ UK; 6 Data Science, Cardiff School of Technologies, Cardiff Metropolitan University, Western Avenue, Cardiff, CF5 2YB UK; 7 Population Data Science, Swansea University, Swansea, Wales SA2 8PP UK; 8 Children’s Social Care Research and Development Centre (CASCADE), School of Social Sciences, SPARK, Cardiff University, Cardiff, CF24 4HQ UK; 9 SPARK, Cardiff University, Cardiff, Wales CF24 4HQ UK; 10 Centre for Health Data Science, University of Aberdeen, Polwarth Building, Foresterhill, AB25 2ZD UK

**Keywords:** Children in need, Children in care, Adversity, Education attainment, Public health, Hospital admissions, Administrative data, Data linkage, Routinely collected data

## Abstract

**Background:**

Research consistently finds poorer health and educational outcomes for children who have experienced out-of-home care relative to the general population. Few studies have explored differences between those in care and those in receipt of intervention from social services but not in care. Children receiving social services interventions often experience Adverse Childhood Experiences (ACEs), and deprivation, which are known to negatively impact outcomes. We aimed to estimate the association of different social services interventions with educational outcomes and hospital admissions, while adjusting for ACEs and deprivation.

**Methods:**

We linked retrospective, routinely collected administrative records from health, education, and social care to create a cohort via the Secure Anonymised Information Linkage (SAIL) databank in Wales, UK. We analysed data for children and household members (*N* = 30,439) across four different groups: (1) no social care intervention; (2) children in need but not in care (CIN); (3) children on the Child Protection Register but not in care (CPR); (4) children in care - i.e. removed from the family home and looked after by the local authority (CLA). Our primary outcome was education outcomes at age 16 years. Secondary outcomes were all cause emergency hospital admissions, and emergency admissions for external causes/injuries.

**Results:**

Children in receipt of social services intervention were more likely to not attain the expected level upon leaving statutory education at age 16 after adjusting for ACEs and other characteristics (for children who had been in out-of-home care (conditional OR: 1·76, (95%CI) 1·25 − 2·48), in need (2·51, 2·00–3·15) and those at risk (i.e., on the child protection register) (4·04, 2·44 − 6·68). For all-cause emergency admissions, all social care groups were at greater risk compared to children in the general population (children in care (conditional HR: 1·31, 1·01–1·68), children in need (1·62, 1·38 − 1·90), and children at risk (1·51, 1·11 − 2·04).

**Conclusions:**

All groups receiving social service intervention experience poorer educational and health outcomes than peers in the general population. Children who remain with their home parents or caregivers but are identified as ‘in need’ or ‘at risk’ by social care practitioners require further research. Integrated support is needed from multiple sectors, including health, educational and social care.

**Supplementary Information:**

The online version contains supplementary material available at 10.1186/s12889-024-20204-6.

## Background

Social service intervention is often provided to families who are experiencing challenges, including familial harm, disability, and challenges in providing parental care. Among families receiving support, some children may be placed into institutional care; an estimated 5.37 million children reside in these settings worldwide [1]. In the UK, three broad categories of intervention can be identified. One is removal from parental care and placement in ‘out-of-home’ care (or ‘in care’, the commonly used shorthand). This group will henceforth be referred to as children ‘looked after’, or CLA, with reference to the UK legal term. Being looked after by a local authority most commonly involves foster care, most typically by alternative carers who are not related to the children. However, sometimes care by relatives or family friends (kinship care) can be classed as foster care. Then a minority of CLA live in residential care, typically with a small group of other children and a range of staff on rotation. A second category of children are those who remain within the family home but are identified as at high risk and placed on the child protection register (henceforth, CPR). This is a confidential list that can be consulted by approved professionals (e.g., social workers, teachers and police) to identify children who are at risk. Children on the CPR should be regularly monitored by social services. There will be a plan for reducing risk to the child, that will typically state what changes are expected from the child’s carers and outline the help offered by services. A third group are children identified as in need but not at risk, who are not placed on the CPR but defined as children in need (CIN). This is a heterogeneous group, including children whose families are struggling to care for them and families with children with disabilities who seek respite.

CLA represent 1.1% of all children in Wales [2], whereas children in need, referred to as ‘receiving care and support’, represent 2.6% [3] with 14% of these children on the child protection register [4]. The number of children being looked after is increasing, particularly in Wales, with a 25% increase since 2014. There is considerable variation in the type of help received by these children and their families. In the CIN and CPR groups, there may be, for example, referral to parenting programmes. Some material support may also be offered to families. Children from any of the three groups may be offered therapeutic support. But there is no set offer of services for any one group. The key distinctions between the groups are that children who are CLA have at some point been placed in alternative care and the other two groups have not; while children on the CPR should receive a higher level of monitoring, on the basis of risk, compared to CIN.

A wealth of research shows that children whose families receive social service intervention have lower educational attainment [5–10] and poorer health outcomes [11–16] on average, compared to the general population. Additional risk factors for attainment include short-length of care or instability, special educational needs, being male, of a marginalised ethnicity, socioeconomic deprivation, low expectations, and school movement [6, 9, 10, 17–22]. In terms of health outcomes, a study from Scotland found that looked-after children have 5.5 times higher mortality and experience more health events than children in the general population [23]. These findings are corroborated in other countries across different care types and welfare and social care systems [24, 25]. Studies have also found that the mental health of CLA or CIN is considerably poorer than children with no social service intervention [11, 14, 26–29], and they are at increased probability of risky sexual behaviours [30], and health-harming behaviours, e.g., smoking [29, 31].

Most children who receive social service intervention have previously experienced some, or multiple forms of Adverse Childhood Experiences (ACEs), including abuse, maltreatment, domestic violence, parental substance use or illness [32], which commonly intersect with deprivation or poverty [33]. Several studies have shown that ACEs are associated with lower educational attainment [34–36], and health outcomes [36–39]. While numerous cross-sectional studies suggest that CLA have fewer positive outcomes than the general population, it remains unclear to what extent these outcomes are influenced by care-related factors (such as trauma from removal from families, or poor quality foster or residential care), or the experience of adversities prior to out-of-home care. There is a critical need for research that captures educational and health outcomes of CLA, CIN and CPR children while accounting for ACEs, which likely act as causes of both contact with social services and of poorer educational and health outcomes. Further, there is emerging evidence of better educational outcomes for children who are in care, by comparison to those in receipt of social service intervention but not in care [10], suggesting a potentially protective effect of out-of-home care for some children.

In this study, we used record-linked routine administrative healthcare, social care, and education data to explore hospital admissions and education outcomes for children looked-after (CLA), in need (CIN) and on the child protection register (CPR). Our study was designed around a primary outcome of educational attainment, but taking a multidisciplinary approach, recognising the interconnectedness of education and health outcomes and their drivers throughout the life course [40], we analysed emergency healthcare admissions as a secondary outcome.

## Methods

### Aim, design and setting of the study

Our study had three overarching aims:

1. To examine if type of social services intervention (CIN, CPR, and CLA) is associated with worse health and education outcomes compared who children who did not receive social care intervention in Wales, UK;
2. To explore the extent to which the association between social service intervention and outcomes is explained by prior childhood experiences (ACEs);
3. To estimate whether social service intervention moderates the association between ACEs and the outcomes.

**Fig. 1:**
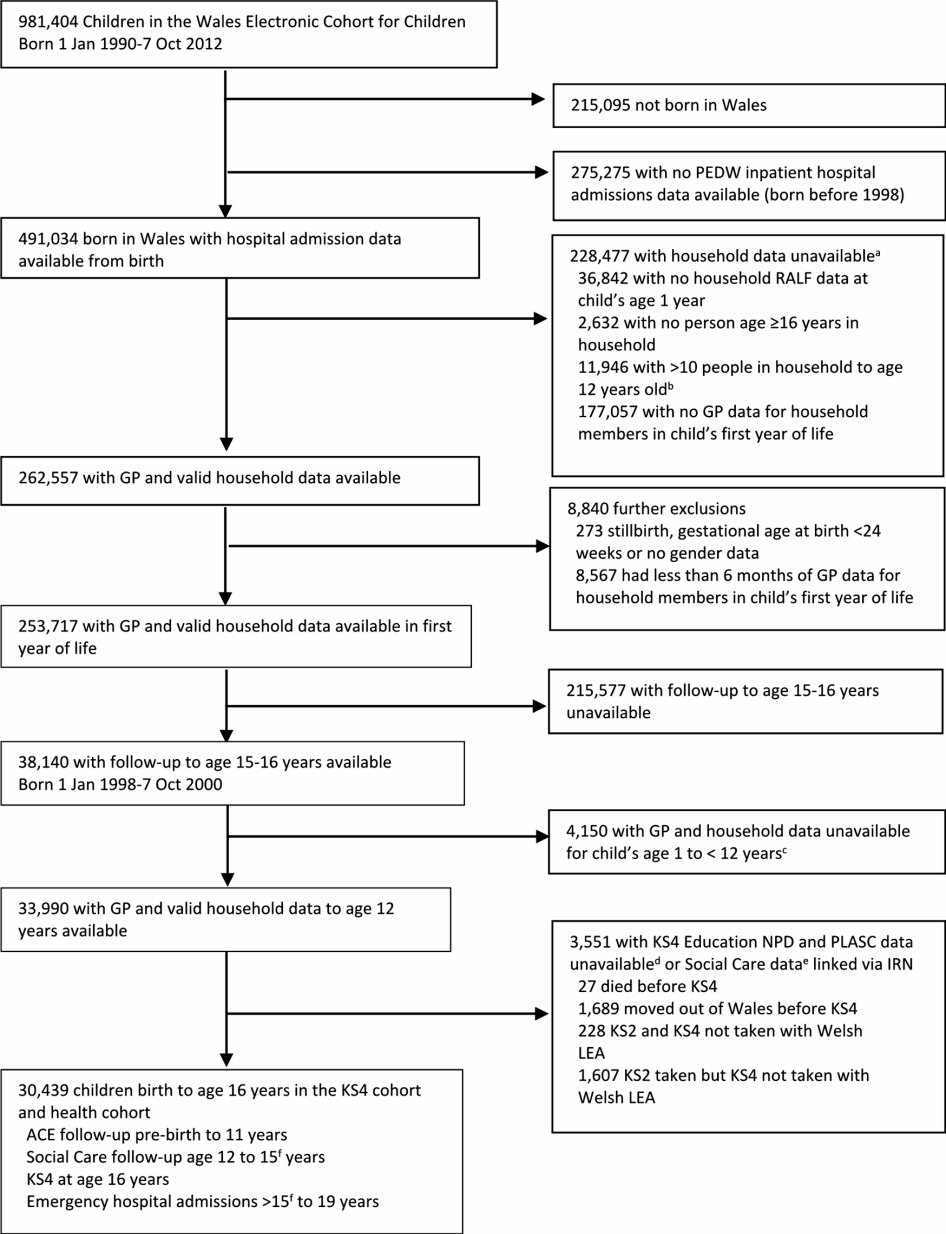
Anonymised participant selection for analyses *PEDW = Patient Episode Database Wales; RALF = Residential Anonymous Linking Field; GP = General Practice; IRN = Individual Registration Number; KS = Key Stage; PLASC = Pupil Level Annual School Census; LEA = Local Education Authority;*^*a*^*Except for children who died (n = 1686) or moved out of Wales (n = 5020) in the first year of life.*^*b*^*The Unique Property Reference Number is considered inaccurate if there are more than ten people in a household;*^*c*^*6 months of GP data available for at least one household member for child’s age time windows 1 to < 5 years*,* 5 to < 8 years*,* 8 to < 12 years;*^*d*^*independent schools*,* severely disabled children who are not catered for by Special Educational Needs provision in the LEA school system*,* those outside administrative systems e.g. travellers;*^*e*^*Children in Need (CIN) Census and Looked after children Wales (LACW) dataset;*^*f*^*age 15 years at CIN Census date*

We used a population-based e-cohort, the Welsh Electronic Cohort of Children (WECC), and linked this with routine data on health, education and social services from the Secure Anonymised Information Linkage Databank (SAIL), based at Swansea University in Wales, UK [41–44]. WECC is a subset of SAIL and holds data on all children born and living in Wales from 1990 [34, 39, 45]. Study entry (Fig. 1 depicts inclusion and exclusion information) is defined by [1] being born in Wales with a week of birth in 1st Jan 1998–7th Oct 2000, and [2] available primary care consultations data, also known as General Practice data, for adult household members to children aged 12 years (via residential address), and [3] educational attainment data with the Local Education Authority (LEA) at Key Stage 4 (KS4) at age 16 years. Educational records were linked from the National Pupil Database, which contains information relating to pupil demography, attendance, and educational attainment. Health records were linked inclusive of the Patient Episode Database for Wales and the Welsh Longitudinal General Practice dataset. The Patient Episode Database for Wales includes inpatients and day-cases, with demographic and clinical information on hospital admissions, including primary diagnoses and co-morbidities; the Welsh Longitudinal General Practice dataset includes Read codes (a set of clinical terms in the UK) for symptoms, diagnoses, and prescriptions.

### Participants

To identify children in receipt of social service intervention, the WECC cohort was linked to the education dataset first (via Individual Reference Number in Wales), and then to the Children in Need census (CIN) for children who are CIN/CPR, and we used a ‘looked after flag’ variable in the CIN to identify looked after children (CLA). A child is deemed having been ‘looked after’ if they have been provided with accommodation by the local authority (small areas of governance in Wales), for more than 24 h, or placed in the care of a local authority. It is important to note that the data used to identify CIN only represented 80% of all CIN as they had to have a case open for ≥ 3 months; following this, the CIN dataset was no longer operational after 2015-16, and now the Children Receiving Care and Support dataset (CRCS) is in place. Consequently, a small number of young people will be misclassified as part of the “not in receipt of social service intervention” comparator (around 1%).

## Measures

### Exposures

*Child care status*: Each child was classified into their highest category of need at any time during the ages of 12 to 15 years with categories: None, CIN, CPR, CLA; for example, a child who was classified as CIN at age 12 years and CPR at age 13 years would be in the CPR category. Categories of social service intervention (CIN/CPR/CLA) were derived from the CIN Census data (2010–2015) for a child between the age of 12 to 15 years, or general population (those not in the CIN dataset categorised as ‘None’). CLA and CPR represent subsets of CIN, while a third category included CIN who were neither CPR nor CLA.

#### Adverse childhood experiences (ACEs)

Within the WECC, we have previously coded five adverse childhood experiences using the Patient Episode Database for Wales and the Welsh Longitudinal General Practice dataset [34, 39, 45]. These include childhood victimisation resulting in a hospital admission (physical abuse); alcohol-related hospital admission in an adult in the household (parental substance use), derived using our published method [46], and a General Practice (GP) record of poor mental health for an adult living in the household (parental mental health illness) - this is separated into Common Mental Disorders (CMD, e.g. depression, anxiety) or serious mental illness (e.g. psychosis). A history of either CMD, serious mental illness or alcohol problems were defined using data from 1st January 1998 when hospital inpatient admissions and GP data were available. Children’s ACEs were measured before the child’s birth up to less than 5 years, and 5 years to less than 12 years. Reference categories in models for ACEs are where a child has no exposure recorded.

### Outcomes

#### Education

The primary outcome are binary indicators of educational attainment at Key Stage 4 (age 15/16 years, the age at which children in the UK leave statutory education). We developed a binary variable indicating that children had met the expected level inclusive of English or Welsh and Mathematics (Grade C or above) vs. not met the expected level.

#### Emergency admissions

We investigated first emergency admissions for all-causes combined, and separately for injuries and external causes; the latter may be associated with inconsistent care. The follow-up for these outcomes was from age 15 years on the day after the CIN Census date of the 31 March to age less than 20 years.

### Covariates

Birth, demographic, and school characteristics (education models only) were used as covariates in the models. Birth characteristics included congenital anomalies (none, minor, major) maternal smoking (yes/no), gestational age (24 - < 33 weeks, 33 weeks - <37 weeks and > 37 weeks), and whether the child was small for their gestational age (yes/no). Demographic characteristics included gender of the child (male/female) and Townsend index of deprivation at birth in quintiles (5 = most deprived). School characteristics included school absence (0, 1–5, 6–10, 11–16 and 17 + days), number of schools attended (1–2, 3, 4, 5, 6+) and academic season of birth (September – December, January – April, May – August). Also included was Free School Meal (FSM) entitlement at KS1 or KS2 (aged 6–7 and 10/11 years), where children whose parents receive financial support from the government receive free meals at school (yes/no). Special Educational Needs (SEN) provision was also included and depicts children who require support at school for disabilities or behavioural problems and receive additional support in order of severity (None, School Action, School Action+, Statemented).

### Statistical analysis

Analyses were conducted on Stata version 17 [47]. Descriptive analysis was used to explore variable distributions and bivariate associations. For educational outcomes (did not attain expected level as the reference category), multilevel logistic regression (QR decomposition) grouped by schools was used; this model allows for children’s unobserved shared factors leading to correlation of educational outcomes within schools when estimating the standard errors for effect sizes. Educational attainment models are interpreted using unadjusted (marginal) Odds Ratios (OR) and conditional Odds Ratios (cOR) that are conditional on other covariates included in the model. For health outcomes, Cox regression was used for time to first emergency hospital admission/external causes or injury at age of 15 years on the day after the CIN Census date of 31 March to less than age 20 years, with censoring for death or migration out of Wales; estimates of these models are interpreted using unadjusted (marginal) Hazard Ratios (HR), and conditional Hazard Ratios (cHR). A fully adjusted model with potential risk factors of ACEs and other potential confounders was then fitted; proportional conditional hazards assumptions were checked. Both education and health models were estimated with (i) ACEs, adjusted for demographic and birth characteristics (objective 2), (ii) social care intervention, adjusted for demographic and birth characteristics (objective 1), (iii) ACEs, social care intervention, adjusted for all characteristics (objective 3), and (iv) a subset of children with social care intervention only, adjusted for all measured characteristics. We investigated interactions between ACEs and social care intervention in both the education and health outcome models. For the education model we checked model fit using the Hosmer Lemeshow goodness-of-fit test and used likelihood ratio tests to assess interactions.

As the study variables have missing data (specifically the confounders; see supplementary material Table 1), we obtained estimates using multiple imputation by chained equations [48] that ignored the multilevel structure of the education models. We included every other variable in the imputation model for each incomplete variable, drawing five proper imputations for each missing value, with each imputation drawn following 10 burn-in iterations, and derived pooled estimates using Rubin’s rules. The mean, standard deviation, minimum and maximum values of variables were compared between imputed datasets and the original unimputed dataset and showed very little difference between these measures. The same imputed data sets were used for both the education and health outcomes modelling. Directed acyclic graphs [49] were used to visualise causal relationships and aid selection of potential confounder variables in analysis by plotting theoretical a priori cause-and-effect relationships from previous research [50, 51]; see supplementary material Fig. 1.

## Results

### Study sample

30,439 children had data available on educational and health outcomes; for full demographics of the sample see Supplementary Table 1. Approximately 1,189 children received some social service intervention in the time-period analysed; 1% of children were deemed as looked-after (CLA), 2.3% were CIN, and 70% of CIN were on the child protection register (CPR) which is 1.6% overall. Children who had received social care intervention were overrepresented in the most deprived quintile (44% vs. 24.7%), major congenital anomalies (7.9% vs. 4.2%), and among children with Special Educational Needs; for instance, 18.8% of children with social care intervention had been statemented at KS2 compared to 2.9% of children in the general population.

### Educational outcomes

Table 1 shows multilevel logistic regression for Social Care intervention between 12 and 15 years, Adverse Childhood Experiences to age 12 years, and not attaining Key Stage 4 (inc. language and mathematics). In unadjusted models, compared to children not in receipt of social service interventions (objective 1), children in receipt of all forms of social service intervention were substantially more likely not to have achieved the expected level, with ORs for CLA estimated at 5·84 times greater odds (95%CI 4·37 − 7·80), then CIN 6·14 (5·06–7·47), and CPR 9·28 (5·93 − 14·51). In the fully adjusted model (Table 1) (objective 2), all forms of social service intervention remained significantly associated with greater risk of non-attainment, relative to children not in receipt of social service interventions, although the estimated conditional odds ratios were substantially attenuated with CLA estimated at cOR 1·76 (1·25 − 2·48), CIN 2·51 (2·00–3·15), and CPR 4·04 (2·44 − 6·68). These estimates differed only marginally from models prior to adjustment for ACEs. When restricted to children in receipt of social service intervention only, with CIN as the reference category (objective 3), there was some indication of a greater odds of non-attainment among those on the CPR relative to those in need but not on the CPR, and of lower odds of non-attainment among CLA relative to those in need but not on the CPR. However, confidence intervals for both estimates intersected the null. ACEs were associated with higher odds of KS4 non-attainment when in closer proximity to the time when KS4 exams were taken. Models (Table 2) that used multiple imputation were similar to those with no answer categories included in the models; see supplementary material Table 2 for all characteristic cORs.

**Table 1:** The associations between social care intervention and ACEs and educational attainment

	Total / Not attained KS4 Level 2 (inclusive) (%)	Unadjusted OR (95 CI)	Multivariable: ACEs adjusted for other variables^a^ cOR (95% CI)	Multivariable: Social Care adjusted for other variables^a^ cOR (95% CI)	Multivariable: ACEs & Social Care adjusted for other variables^a^ cOR (95% CI)	Social care data only Multivariable: ACEs & Social Care adjusted for other variables^a^ cOR (95% CI)
**N**	30,439 / 12,064 (40)	30,439	30,439	30,439	30,439	1,189
Highest level of Social Care intervention age 12 to 15 years (ref = None)	29,250 / 11,094 (38)	-	-	-	-	-
Other children in need (%)	714 / 579 (81)	6.14 (5.06–7.47)	-	2.59 (2.07–3.25)	2.51 (2.00-3.15)	-
Child protection register (%)	172 / 149 (87)	9.28 (5.93–14.51)	-	4.20 (2.54–6.96)	4.04 (2.44–6.68)	1.56 (0.88–2.74)
Children looked after: out-of-home care (%)	303 / 242 (80)	5.84 (4.37–7.80)	-	1.86 (1.32–2.62)	1.76 (1.25–2.48)	0.71 (0.46–1.10)
Ever a potential child adversity to age 11 years:						
A victimisation hospital admission = yes (%)	294 / 191 (65)	2.68 (2.09–3.44)	1.25 (0.92–1.70)	-	1.17 (0.86–1.60)	0.76 (0.35–1.62)
Household member with serious mental illness = yes (%)	404 / 201 (50)	1.49 (1.22–1.83)	0.83 (0.64–1.07)	-	0.81 (0.62–1.05)	0.91 (0.37–2.22)
A change to a single adult household = yes (%)	7,249 / 3,492 (48)	1.49 (1.41–1.57)	0.99 (0.92–1.06)	-	0.98 (0.92–1.05)	0.76 (0.53–1.09)
Death in the household child age 1 to 11 years = yes (%)	2,143 / 1,010 (47)	1.37 (1.25–1.50)	1.04 (0.93–1.16)		1.03 (0.92–1.15)	0.82 (0.49–1.39)
Household member with a common mental disorder						
History to < 5 years = yes (%)	9,337 / 4,223 (45)	1.36 (1.29–1.44)	1.03 (0.96–1.10)	-	1.03 (0.96–1.10)	0.69 (0.47-1.00)
5 to < 12 years = yes (%)	12,582 / 5,724 (46)	1.47 (1.40–1.54)	1.14 (1.07–1.21)	-	1.13 (1.06–1.21)	1.07 (0.72–1.57)
Household member with an alcohol problem						
History to < 5 years = yes (%)	2,713 / 1,505 (56)	1.87 (1.72–2.03)	1.09 (0.98–1.21)	-	1.06 (0.96–1.19)	1.26 (0.80-2.00)
5 to < 12 years = yes (%)	4,115 / 2,269 (55)	1.93 (1.80–2.07)	1.27 (1.16–1.38)	-	1.25 (1.15–1.36)	1.29 (0.86–1.95)
Free school meals eligible^b^ (ref = No)	23,692 / 7,608 (32)	-	--	--	--	--
Persistent: at KS1 & KS2 (%)	3,166 / 2,238 (71)	4.49 (4.13–4.88)	2.30 (2.07–2.55)	2.28 (2.06–2.53)	2.17 (1.96–2.41)	1.66 (1.06–2.60)
At KS1 and not KS2 (%)	1,609 / 985 (61)	3.01 (2.70–3.35)	1.80 (1.58–2.04)	1.82 (1.61–2.06)	1.77 (1.56–2.01)	1.45 (0.77–2.70)
At KS2 and not KS1 (%)	1,601 / 962 (60)	2.87 (2.58–3.19)	1.73 (1.52–1.96)	1.75 (1.55–1.99)	1.69 (1.49–1.92)	1.59 (0.88–2.89)
Townsend deprivation quintile at birth^b^ (ref = 1 – least dep)	4,968 / 1,133 (23)	-	-	-	-	-
5 – most (%)	7,531 / 4,044 (54)	2.98 (2.72–3.25)	1.69 (1.52–1.88)	1.71 (1.54–1.90)	1.69 (1.52–1.88)	1.16 (0.62–2.19)
Number of schools attended (ref = 1 to 2)	15,860 / 5,606 (35)	-	-	-	-	-
6+ (%)	291 / 203 (70)	3.47 (2.66–4.51)	2.02 (1.49–2.74)	1.87 (1.38–2.54)	1.79 (1.32–2.44)	3.43 (1.32–8.91)
Number of days absent in year take KS2^b^ (ref = None)	3,542 / 1,185 (34)	-	-	-	-	-
17+ (%)	6,653 / 3,662 (55)	2.36 (2.16–2.58)	1.67 (1.50–1.85)	1.72 (1.55–1.90)	1.68 (1.51–1.86)	1.27 (0.70–2.29)

^a^ school year (reference year 2015), sex, gestational age at birth, small for gestational age (< 10th centile), academic season of birth, congenital anomaly, maternal age at childbirth, maternal smoking in first trimester, deprivation quintile at birth, Special Educational Needs provision (SEN) at KS1, SEN at KS2, Free School Meals eligible at KS1 or KS2, number of schools attended, number of days absent in year take KS2; see Supplementary Table 2 for model results for other variables^a^; ^b^ <5% missing data

**Table 2:** Time until hospital admission (> 15 years to < 20 years) post-social care intervention (*N* = 30,439)

	Proportion (%)	Unadjusted HR (95% CI)	Multivariable: ACEs adjusted for other variables^a^ cHR (95% CI)	Multivariable: Social Care adjusted for other variables^a^ cHR (95% CI)	Multivariable: ACEs & Social Care adjusted for other variables^a^ cHR (95% CI)	Social care data only Multivariable: ACEs & Social Care adjusted for other variables^a^ cHR (95% CI)
N	30,439	30,439	30,439	30,439	30,439	1,189
Highest level of Social Care intervention age 12 to 15 years (ref = None)	3836 (93)	-	-			
Other children in need (%)	178 (4)	2.02 (1.74–2.35)	-	1.67 (1.42–1.95)	1.62 (1.38–1.90)	
Child protection register (%)	43 (1)	2.07 (1.53–2.79)	-	1.54 (1.14–2.10)	1.51 (1.11–2.04)	0.99 (0.70–1.41)
Children looked after: out-of-home care (%)	65 (2)	1.77 (1.38–2.26)	-	1.37 (1.07–1.76)	1.31 (1.01–1.68)	0.78 (0.57–1.07)
Ever a potential child adversity to age 11 years:				-		
A victimisation hospital admission = yes (%)	60 (2)	1.61 (1.25–2.08)	1.28 (0.99–1.66)	-	1.23 (0.95–1.60)	1.37 (0.84–2.23)
Household member with serious mental illness = yes (%)	73 (2)	1.38 (1.10–1.75)	1.12 (0.88–1.41)	-	1.10 (0.87–1.39)	1.44 (0.85–2.43)
A change to a single adult household = yes (%)	1148 (28)	1.25 (1.17–1.34)	1.07 (1.00–1.15)	-	1.07 (0.99–1.15)	0.84 (0.65–1.09)
Death in the household child age 1 to 11 years = yes (%)	338 (8)	1.18 (1.06–1.32)	1.08 (0.96–1.21)		1.07 (0.96–1.20)	0.99 (0.68–1.44)
Household member with a common mental disorder		-		-		
History to < 5 years = yes (%)	1423 (35)	1.22 (1.14–1.30)	1.09 (1.01–1.17)	-	1.09 (1.01–1.17)	1.31 (1.01–1.68)
5 to < 12 years = yes (%)	1897 (46)	1.22 (1.15–1.30)	1.11 (1.04–1.19)	-	1.11 (1.03–1.18)	0.82 (0.64–1.07)
Household member with an alcohol problem		-		-		
History to < 5 years = yes (%)	445 (11)	1.28 (1.16–1.42)	1.03 (0.93–1.15)	-	1.02 (0.91–1.13)	1.19 (0.90–1.59)
5 to < 12 years = yes (%)	680 (17)	1.27 (1.17–1.38)	1.06 (0.97–1.16)	-	1.05 (0.96–1.14)	0.77 (0.58–1.02)
Free school meals eligible^b^ (ref = No)	2906 (71)	-				
Persistent: at KS1 & KS2 (%)	600 (15)	1.58 (1.45–1.73)	1.22 (1.10–1.35)	1.23 (1.11–1.36)	1.17 (1.06–1.30)	0.92 (0.67–1.26)
At KS1 and not KS2 (%)	285 (7)	1.46 (1.29–1.65)	1.20 (1.05–1.36)	1.23 (1.08–1.40)	1.18 (1.04–1.35)	1.23 (0.80–1.90)
At KS2 and not KS1 (%)	247 (6)	1.29 (1.13–1.46)	1.05 (0.91–1.20)	1.07 (0.94–1.23)	1.03 (0.90–1.18)	1.41 (0.96–2.06)
Townsend deprivation quintile at birth^b^ (ref = 1 – least deprived): 5 – most (%)	1,152 (28)	1.39 (1.26–1.54)	1.08 (0.97–1.21)	1.09 (0.98–1.21)	1.08 (0.97–1.21)	0.85 (0.54–1.34)

^a^ sex, gestational age at birth, small for gestational age (< 10th centile), academic season of birth, congenital anomaly, maternal age at childbirth, maternal smoking in first trimester, Townsend deprivation quintile at birth, Special Educational Needs provision (SEN) at KS1, SEN at KS2, Free School Meals eligible at KS1 or KS2, number of schools attended, number of days absent in year take KS2; see Supplementary Table 3 for model results for other variables^a^; ^b^ <5% missing data

### Health outcomes

Table 2 shows the results from a Cox regression for Social Care intervention between age 12 and 15 years, exposure to Adverse Childhood Experiences to age 12 years and time to first all-cause emergency admission after age 15 years on 31st March (Social Care Census date) and < 20 years. The full model with all covariates is shown in Table 3 of the supplementary material. In unadjusted models, children in receipt of all forms of social service intervention (objective 1) were substantially more likely to have experienced hospital admission with estimated HRs with CLA at a 1·77 greater risk (95%CI 1·38 − 2·26), CIN at 2.02 (1·74 − 2·35) and CPR being 2·07 (1·53 − 2·79) compared to no intervention. In the fully adjusted model (objective 2), all forms of social service intervention remained significantly associated with greater risk of admission, relative to the general population sample, although the estimated conditional hazard ratios were substantially attenuated: for CLA this was cHR 1·31 (1·01–1·68), CPR was estimated at 1·51 (1·11 − 2·04), and CIN at 1·62 (1·38 − 1·90). However, estimates only differed marginally from models prior to adjustment for ACEs. Where restricted to children in receipt of social service intervention, with CIN as the reference category (objective 3), there was some indication of lower risk of admission among CLA. However, confidence intervals were wide and intersected the null.

**Table 3:** Time to first injury or external cause emergency admission (> 15 years to < 20 years) (*N* = 30,439)

	Proportion (%)	Unadjusted HR (95 CI)	Multivariable: ACEs adjusted for other variables^a^ cHR (95% CI)	Multivariable: Social Care adjusted for other variables^a^ cHR (95% CI)	Multivariable: ACEs & Social Care adjusted for other variables^a^ cHR (95% CI)	Multivariable: ACEs & Social Care adjusted for other variables^a^ cHR (95% CI) (Interactions)	Social care data Multivariable: ACEs & Social Care adjusted for other variables^a^ cHR (95% CI)
Highest level of Social Care intervention age 12 to 15 years (ref = None)	1071 (91)	-	-	-	-	-	-
Other children in need (CIN) / Child protection register (CPR) (%)	81 (7)	2.56 (2.04–3.21)	-	2.19 (1.72–2.78)	2.09 (1.64–2.66)	2.07 (1.50–2.85)	-
Looked after children Wales (CLA): out-of-home care (%)	31 (3)	2.96 (2.07–4.23)	-	2.53 (1.75–3.66)	2.33 (1.60–3.39)	1.41 (0.77–2.60)	0.71 (0.35–1.43)
Ever a potential child adversity to age 11 years:							
A victimisation hospital admission = yes(%)	18 (2)	1.64 (1.03–2.60)	1.26 (0.79–2.02)	-	1.10 (0.69–1.78)	1.13 (0.70–1.81)	0.84 (0.33–2.12)
Household member with serious mental illness = yes(%)	20 (2)	1.30 (0.83–2.02)	1.02 (0.65–1.60)	-	0.98 (0.63–1.53)	0.98 (0.63–1.53)	0.99 (0.39–2.51)
A change to a single adult household = yes(%)	354 (30)	1.37 (1.21–1.55)	1.14 (1.00–1.30)	-	1.13 (0.99–1.29)	1.13 (0.99–1.29)	0.82 (0.55–1.23)
Death in the household child age 1 to 11 years = yes(%)	115 (10)	1.42 (1.17–1.72)	1.24 (1.02–1.51)		1.23 (1.01–1.49)	1.23 (1.01–1.49)	0.82 (0.55–1.23)
Household member with a common mental disorder	-	-	-	-	-	-	-
History to < 5 years = yes(%)	553 (47)	1.20 (1.07–1.36)	1.05 (0.92–1.20)	-	1.05 (0.92–1.19)	1.02 (0.88–1.17)	1.05 (0.65–1.68)
Interaction: History to < 5 years X Other CIN/CPR = yes(%)	-	-	-	-	-	1.02 (0.65–1.62)	-
Interaction: History to < 5 years = X CLA = yes(%)	-	-	-	-	-	2.52 (1.19–5.34)	2.41 (1.00 -5.79)
5 to < 12 years = yes(%)	227 (19)	1.25 (1.11–1.40)	1.12 (0.98–1.27)	-	1.11 (0.98–1.26)	1.12 (0.98–1.26)	1.11 (0.73–1.69)
Household member with an alcohol problem	-	-	-	-	-	-	-
History to < 5 years = yes(%)	14 (12)	1.47 (1.23–1.74)	1.47 (1.09–1.98)	-	1.06 (0.88–1.28)	1.41 (1.05–1.90)	1.16 (0.57–2.35)
Interaction: History to < 5 years X Time to outcome (per year)	-	-	-	-	-	0.98 (0.96–1.00)	1.00 (0.95–1.05)
5 to < 12 years = yes(%)	227 (19)	1.52 (1.32–1.76)	1.22 (1.05–1.43)	-	1.18 (1.01–1.38)	1.18 (1.01–1.38)	0.75 (0.48–1.17)
Free school meals eligible^b^ (ref = No)	812 (69)	-	-	-	-	-	-
Persistent: at KS1 & KS2 (%)	188 (16)	1.75 (1.48–2.05)	1.32 (1.08–1.61)	1.32 (1.08–1.61)	1.22 (1.00–1.49)	1.22 (1.00–1.49)	1.08 (0.65–1.78)
At KS1 and not KS2 (%)	84 (7)	1.55 (1.24–1.94)	1.25 (0.99–1.58)	1.27 (1.01–1.61)	1.20 (0.95–1.52)	1.21 (0.96–1.53)	1.16 (0.57–2.35)
At KS2 and not KS1 (%)	72 (6)	1.33 (1.04–1.70)	1.05 (0.81–1.36)	1.07 (0.83–1.38)	1.01 (0.78–1.30)	1.01 (0.78–1.31)	1.22 (0.64–2.32)
Townsend deprivation quintile at birth^b^ (ref = 1 – least deprived): 5 – most (%)	339 (29)	1.48 (1.22–1.80)	1.09 (0.89–1.34)	1.10 (0.90–1.35)	1.09 (0.88–1.33)	1.09 (0.89–1.34)	0.86 (0.41–1.79)

^a^ sex, gestational age at birth, small for gestational age (< 10th centile), academic season of birth, congenital anomaly, maternal age at childbirth, maternal smoking in first trimester, Townsend deprivation quintile at birth, Special Educational Needs provision (SEN) at KS1, SEN at KS2, Free School Meals eligible at KS1 or KS2, number of schools attended, number of days absent in year take KS2; see Supplementary Table 4 for model results for other variables^a^ ;^b^ <5% missing data

Table 3 shows the results from a Cox regression for Social Care intervention between 12 and 15 years, exposure to Adverse Childhood Experiences to age 12 years and time to first injury or external cause emergency admission after age 15 years on 31st March (Social Care Census date). In these models CPR children were merged with CIN due to small numbers. Unadjusted models showed CLA had the highest risk (HR 2·96, 2·07 − 4·23), and CPR/CIN (2·56, 2·04 − 3·21) had a similar risk to CLA, compared to those with no intervention. Once fully adjusted for covariates, both groups differed from the general population with small differences between their estimates (CLA cHR 2·33, 1·60 − 3·39; CPR/CIN 2·09, 1·64 − 2·66). There was evidence of an interaction between ACEs and CLA in this model. Specifically, when comparing the risk of an emergency hospital admission for external causes or injury in CLA compared to those without Social Care intervention, those who had ever lived with someone with CMD between their birth and 5 years were estimated to have a conditional HR 2·52 (1·19 − 5·34) times higher than those with no Social Care intervention. We also found living with someone with an alcohol problem between birth and 5 years interacted with time and the hazard ratio reduced slightly per year after the age of 15 years to less than 20 years. For models adjusted for all covariates, see supplementary material. Where restricted to children in receipt of social service intervention, with CIN/CPR as the reference category, no evidence of an association was found for external cause or injury admissions.

## Discussion

The current study aimed to examine if type of social services intervention (CIN, CPR, and CLA) is associated with worse health and education outcomes compared who children who did not receive social care intervention in Wales, UK. Our analyses found that all groups of children in receipt of social service intervention were more likely to not attain the expected level of education upon leaving statutory education at KS4 (age 16 years), with some suggestion that those on the CPR were at particularly high risk. Given the interconnectedness of the drivers of health and educational outcomes, our analysis also provided novel insight on the increased risk of hospital admissions, both generally and due to external causes and injury for children in receipt of different levels of social service intervention. To our knowledge, this is the first study to explore hospital admissions for children in receipt of social service intervention in the UK; a study in Scotland only appears to focus on CLA health outcomes [23]. The separation of CPR from CIN is a novel and important distinction. Most existing studies have only differentiated CIN and CLA and found CIN children have lower educational outcomes [10]. Educational outcomes are a key predictor of inequalities in life chances, including health inequalities, throughout the life course [40]. The addition of comparing types of social service interventions was suggestive of disparities, and further investigation is required of outcomes for different subgroups of children in receipt of social services intervention.

The second aim of this paper was to explore the extent to which the association between social service intervention and outcomes was explained by prior childhood experiences (ACEs). We found marginal effects of ACEs. However, we were only able to examine a limited number of ACEs and our findings may not reflect findings of low-level chronic ACE’s that do not result in contact with health or social care. We found that previously living with someone with a common mental health disorder (i.e., depression, or anxiety), or an alcohol problem (i.e., via hospital admission or medical consultation) was associated with both reduced educational attainment and increased emergency hospital admissions. Our findings reflect those of Evans et al. (2020) [34], which explored the association between ACEs and educational attainment and found that alcohol and common mental health disorders were significantly associated with reduced education attainment in childhood. In contrast to Evans et al., (2020) we did not find associations between attainment and prior childhood victimisation or serious mental illness, or death in childhood household. In terms of hospital admissions, fewer studies have explored this. Bellis et al. (2017) [52] found that the number of ACEs were positively associated with a greater number of health practitioner visits, emergency department attendance, and overnight stays in hospitals adjusted for deprivation. Wider research finds that physical health outcomes are lower among those who have a greater number of ACEs and for specific ACEs (e.g., abuse, neglect) [53]. However, we did not find strong evidence that the association of social service intervention with educational or health outcomes was confounded by ACEs as measured in this study, with estimates only marginally altered in models which adjusted for ACEs relative to those which did not.

The third aim was to estimate whether social service intervention moderates the association between ACEs and the outcomes. We found limited interaction; however, early exposure of living with someone with a common mental health problem (< 5 years old) and being looked-after (CLA) was associated with higher hospital admission compared to those without experience of social service intervention. Counterintuitively, it may be expected that being in care would offer a protective factor against hospital admissions. Broader research in this area suggests that mothers with higher depressive symptoms engage in fewer ‘intense’ periods of supervision of children, potentially leading to more opportunities for hazards, and/or development of risky behaviours amongst children, and thus hospital admissions later on [54].

Our results have important implications for researchers, practitioners, and policymakers across education, healthcare, and social services. Adopting a multidisciplinary approach, this study examined both hospital admissions and education outcomes, and observed that all groups of children in receipt of intervention have elevated risk when compared to the general population. Children in contact with social services may benefit from a collective and integrated policy and practice response across sectors to improve outcomes. Of the children who receive help from social services, policy priority within the wider system tends to focus on CLA, for example medical check-ups and additional education support for this group. Our study suggests that CIN and on CPR warrant at least as much policy attention and investment. Investing in more upstream interventions may reduce the burden and economic costs of not acting, including by preventing entry to care and reducing the likelihood of lower academic attainment and increased negative healthcare outcomes [55]. Furthermore, given that ACEs and deprivation are likely pathways towards becoming CPR, CIN and CLA, interventions which successfully reduce these harms and support families should be prioritised. Governments and institutions that incorporate the ‘Health in All Policies’ (HiAP) approach aim to ensure that all sectors incorporate considerations of health and wellbeing in their decisions, along with considerations of equality, equity and sustainability [56].

### Strengths and limitations

Our study had several strengths, but also some important limitations. We were able to identify social service intervention between the ages of 12–15 years in our birth cohort, thus children included in this study may have historic social services intervention that was not captured. Longitudinal research on care histories in England shows that over half of children (58%) who entered care were only looked after for less than a year, and many were in early childhood, but around a fifth (17.6%) had their first entry as an adolescent [57], suggesting we likely have underrepresented the number of children who had ever received a social service intervention. Data on children in receipt of social services has been limited in Wales; although improvements in data collection are planned [58]. Administratively collected records of victimisation, death and serious mental illness are rare and represent the extremities of events. Indeed, for all exposures we are limited by identification from healthcare and educational services, which is likely to underrepresent the true prevalence. We were not able to differentiate the length of stay in care, but we recognise that this is an important contributor to educational outcomes [8], and we were unable to remove ‘short-breaks’, i.e., where children spend a very short time in local authority care. Hence, associations are likely to be a median point between long and short-term CLA children. In addition, we were unable to estimate escalation, i.e., being a child in need who then becomes looked-after, and some children will have received multiple exposures to different types of care. Some results could be explained by unmeasured confounders – for example, many children with cancer have neither congenital conditions nor special education needs, so would not have been marked as disabled in our analyses but will be classed as ‘in need’ and are likely to have more frequent emergency hospital treatment. Overall, we faced challenges in the use of administrative data, leading us to the conclusion that improvements are needed to administrative data collection systems to support their use in research and evaluation. We also call for supplementation of administrative data with survey and trial data.

## Conclusion

All groups of children in receipt of social services intervention are at risk for higher hospital admissions and lower educational attainment compared to children with no experience of social care intervention, even after allowing for the increased risk of these outcomes associated with exposure to adverse childhood experiences. There was some evidence of differences between social care groups, however this was not clear and requires further investigation. In conclusion, when considering the health and wellbeing needs of children receiving social services intervention, there is a need for policy and practice to focus on all three groups, not just those in out-of-home care, to ensure the needs of those still at home are properly met. We recommend a multi-disciplinary policy and practice approach for supporting children living with their birth families, as well as those in care.

### Patient and public involvement

We engaged with a group of care-experienced young people (CASCADE Voices) throughout on a number of areas: the aims, design, results, conclusion and non-academic outputs of the study. We also had an advisory group which provided advice on the direction and contextualisation of the research. Membership included local authority Heads of Childrens’ Services in Wales, social work practitioners, academics, Welsh Government bodies, as well as representation from health and education practice. We also created an animation to present study results to practitioners from health, social care, and education.

## Electronic supplementary material

Below is the link to the electronic supplementary material.

Supplementary Material 1

## Data Availability

The data that support the findings of this study are available from the Secure Anonymised Information Linkage (SAIL) Databank but restrictions apply to the availability of these data, which were used under license for the current study, and so are not publicly available. Data are however available from the SAIL (https://saildatabank.com/contact/) upon reasonable request with appropriate resource, and with permission of the lead author.
