# Supplementary material for: Receipt of social services intervention in childhood, educational attainment and emergency hospital admissions: longitudinal analyses of national administrative health, social care, and education data in Wales, UK
Source: BMC Public Health. 2024 Oct 21;24:2912. doi: 10.1186/s12889-024-20204-6 (PMC11494773; doi:10.1186/s12889-024-20204-6)
Supplement: Supplementary file 1 — Supplementary Material 1 [file 12889_2024_20204_MOESM1_ESM.docx]

**Supplementary Material**


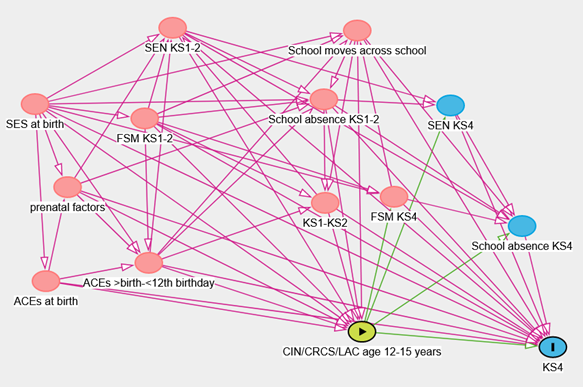


Supplementary Figure 1: Directed Acyclic Graph of theoretical a priori pathways to school attainment via characteristics, ACEs, and social care intervention. In the plot pink circles indicate potential confounder variables, blue circles indicate variables potentially on the causal pathway (mediators), directional arrows show a theoretical cause-and-effect relationship; green circle with black triangle is the exposure; blue circle with vertical black line is the outcome.

**Supplementary Table 1: Characteristics of cohort**

|  | **Total**  **(%)** | **Any Social Care intervention age 12 to 15 years (%)** | **Not attained KS4 Level 2 (language and mathematics) (%)** | **First all-cause emergency admission 15^a^ - < 20 years (%)** | **First injury or external cause emergency admission 15^a^ - < 20 years (%)** |
| --- | --- | --- | --- | --- | --- |
| N | 30,439 | 1,189 | 12,064 (39.6) | 4,122 (13.5) | 1,183 (3.9) |
| Sex=male (%) | 15,584 (51.2) | 695 (50.9) | 6,800 (56.4) | 1,767 (42.9) | 640 (54.1) |
| Townsend deprivation quintile at birth^b^: 1 - least (%) | 4,968 (16.3) | 91 (8) | 1,133 (9.4) | 541 (13.1) | 147 (12.4) |
| 2 (%) | 5,518 (18.1) | 113 (10) | 1,747 (14.5) | 696 (16.9) | 209 (17.7) |
| 3 (%) | 6,039 (19.8) | 187-192 (16) | 2,309 (19.1) | 795 (19.3) | ~238 (20.3) |
| 4 (%) | 6,230 (20.5) | 270 (23) | 2,775(23.0) | 924 (22.4) | 245 (20.7) |
| 5 - most (%) | 7,531 (24.7) | 523 (44) | 4,044 (33.5) | 1,152 (27.9) | 339 (28.7) |
| Congenital anomaly: No (%) | 28,907 (95.0) | 1,080 (90.8) | 11,368 (94.2) | 3,849 (93.4) | 1,119 (94.6) |
| minor (%) | 240 (0.8) | 15 (1.3) | 108 (0.9) | 43 (1.0) | 10 (0.8) |
| major (%) | 1,292 (4.2) | 94 (7.9) | 588 (4.9) | 230 (5.6) | 54 (4.6) |
| Maternal age at childbirth^b^: <18 (%) | 1,008 (3.3) | 98 (8.2) | 619 (5.1) | 186 (4.5) | 56 (4.7) |
| 18-24 (%) | 8,183 (26.9) | 535 (45) | 4,308 (35.7) | 1,340 (32.5) | 399 (33.7) |
| 25-29 years (%) | 9,245 (30.4) | 284 (23.9) | 3,434 (28.5) | 1,228 (29.8) | 346 (29.2) |
| 30-34 (%) | 8,242 (27.1) | 193 (16.2) | 2,520 (20.9) | 972 (23.6) | 265 (22.4) |
| 35+ (%) | 3,740 (12.3) | 79 (6.6) | 1,173 (9.7) | 391 (9.5) | ~113 (9.8) |
| Academic season of birth: Sep to Dec (%) | 8,170 (26.8) | - | 2,957 (24.5) | - | - |
| Jan to Apr (%) | 10,690 (35.1) | - | 4,235 (35.1) | - | - |
| May to Aug (%) | 11,579 (38.0) | - | 4,872 (40.4) | - | - |
| Gestation at birth^c^: 24-27 (%) | 70 (0.2) | 5 (0.4) | 37 (0.3) | - | - |
| 28-32 (%) | 382 (1.3) | 27 (2.3) | 197 (1.6) | 69 (1.7) | 15 (1.3) |
| 33-36 (%) | 1,703 (5.6) | 92 (7.7) | 728 (6.0) | 260 (6.3) | 60 (5.1) |
| 37+ weeks (%) | 26,425 (86.8) | 982 (82.6) | 10,283 (85.2) | 3,545 (86.0) | 1,022 (86.4) |
| Small for gestational age (<10^th^ centile)^c^=yes(%) | 2,821 (9.3) | 179 (15.1) | 1,412 (11.7) | 429 (10.4) | 107 (9.0) |
| Maternal smoking in first trimester: No (%) | 3,418 (11.2) | 92 (7.7) | 1,126 (9.3) | 415 (10.1) | 122 (10.3) |
| Yes (%) | 1,235 (4.1) | 87 (7.3) | 706 (5.9) | 203 (4.9) | 71 (6.0) |
| NA (%) | 25,786 (84.7) | 1010 (84.9) | 10,232 (84.8) | 3,504 (85.0) | 990 (83.7) |
| SEN provision at KS2^b^: None (%) | 22,356 (73.4) | 468 (39.4) | 5,994 (49.7) | 2,857 (69.3) | 807 (68.2) |
| School action (%) | 4,409 (14.5) | 240 (20.2) | 3,194 (26.5) | 700 (17.0) | 210 (17.8) |
| School action plus (%) | 2,524 (8.3) | 223 (18.8) | 1,974 (16.4) | 395 (9.6) | 121 (10.2) |
| Statemented (%) | 887 (2.9) | 223 (18.8) | 755 (6.3) | 129 (3.1) | 32 (2.7) |
| SEN provision at KS1^b^: None (%) | 22,510 (74.0) | 491 (41.3) | 6,596 (54.7) | 2,920 (70.8) | 831 (70.2) |
| School action (%) | 4,372 (14.4) | 277 (23.3) | 3,031 (25.1) | 659 (16.0) | 199 (16.8) |
| School action plus (%) | 2,009 (6.6) | 205 (17.2) | 1,523 (12.6) | 302 (7.3) | 86 (7.3) |
| Statemented (%) | 494 (1.6) | 130 (10.9) | 393 (3.3) | 69 (1.7) | 18 (1.5) |
| Days absent in year take KS2^b^: None (%) | 3,542 (11.6) | - | 1,185 (9.8) | - | - |
| 1 to 5 (%) | 7,120 (23.4) | - | 2,121 (17.6) | - | - |
| 6 to 10 (%) | 7,139 (23.5) | - | 2,584 (21.4) | - | - |
| 11 to 16 (%) | 5,722 (18.8) | - | 2,365 (19.6) | - | - |
| 17+ (%) | 6,653 (21.9) | - | 3,662 (30.4) | - | - |
| Number of schools to age 15 years: 1 to 2 (%) | 15,860 (52.1) | - | 5,606 (46.5) | - | - |
| 3 (%) | 9,676 (31.8) | - | 3,941 (32.7) | - | - |
| 4 (%) | 3,638 (12.0) | - | 1,754 (14.5) | - | - |
| 5 (%) | 974 (3.2) | - | 560 (4.6) | - | - |
| 6+ (%) | 291 (1.0) | - | 203 (1.7) | - | - |

*Age 15 years on 4th April (day after Social Care Census date); ^b^ <5% missing data; ^c^ <7% missing data

**Supplementary Table 2 - Multilevel logistic regression (Table 1 continued for confounders) for Social Care intervention between 12 to 15 years, Adverse Childhood Experiences to age 12 years and not attaining Key Stage 4 Level 2 (inc. language and mathematics) outcome**

|  | **Total / Not attained KS4 Level 2 (inclusive) (%)** | **Unadjusted**  **OR (95 CI)** | **Multivariable: ACEs adjusted for other variables^a^**  **OR (95% CI)** | **Multivariable: Social Care adjusted for other variables^a^**  **OR (95% CI)** | **Multivariable: ACEs & Social Care adjusted for other variables^a^**  **OR (95% CI)** | **Social care data only**  **Multivariable: ACEs & Social Care adjusted for other variables^a^**  **OR (95% CI)** |
| --- | --- | --- | --- | --- | --- | --- |
| **N** | 30,439 / 12,064 (40) |  |  |  |  |  |
| Highest level of Social Care intervention age 12 to 15 years (ref=None) | 29,250 / 11,094 (38) | 1.00 | - | 1.00 | 1.00 | - |
| Other children in need (%) | 714 / 579 (81) | 6.14 (5.06-7.47) | - | 2.59 (2.07-3.25) | 2.51 (2.00-3.15) | 1.00 |
| Child protection register (%) | 172 / 149 (87) | 9.28 (5.93-14.51) | - | 4.20 (2.54-6.96) | 4.04 (2.44-6.68) | 1.56 (0.88-2.74) |
| Children looked after: out-of-home care (%) | 303 / 242 (80) | 5.84 (4.37-7.80) | - | 1.86 (1.32-2.62) | 1.76 (1.25-2.48) | 0.71 (0.46-1.10) |
| **Ever a potential child adversity to age 11 years:** | | | | | | |
| A victimisation hospital admission=yes (%) | 294 / 191 (65) | 2.68 (2.09-3.44) | 1.25 (0.92-1.70) | - | 1.17 (0.86-1.60) | 0.76 (0.35-1.62) |
| Household member with serious mental illness=yes (%) | 404 / 201 (50) | 1.49 (1.22-1.83) | 0.83 (0.64-1.07) | - | 0.81 (0.62-1.05) | 0.91 (0.37-2.22) |
| A change to a single adult household=yes (%) | 7,249 / 3,492 (48) | 1.49 (1.41-1.57) | 0.99 (0.92-1.06) | - | 0.98 (0.92-1.05) | 0.76 (0.53-1.09) |
| Death in the household child aged 1 to 11 years=yes (%) | 2,143 / 1,010 (47) | 1.37 (1.25-1.50) | 1.04 (0.93-1.16) |  | 1.03 (0.92-1.15) | 0.82 (0.49-1.39) |
| Household member with a common mental disorder |  |  |  |  |  |  |
| History to < 5 years=yes (%) | 9,337 / 4,223 (45) | 1.36 (1.29-1.44) | 1.03 (0.96-1.10) | - | 1.03 (0.96-1.10) | 0.69 (0.47-1.00) |
| 5 to < 12 years=yes (%) | 12,582 / 5,724 (46) | 1.47 (1.40-1.54) | 1.14 (1.07-1.21) | - | 1.13 (1.06-1.21) | 1.07 (0.72-1.57) |
| Household member with an alcohol problem |  |  |  |  |  |  |
| History to < 5 years=yes (%) | 2,713 / 1,505 (56) | 1.87 (1.72-2.03) | 1.09 (0.98-1.21) | - | 1.06 (0.96-1.19) | 1.26 (0.80-2.00) |
| 5 to < 12 years=yes (%) | 4,115 / 2,269 (55) | 1.93 (1.80-2.07) | 1.27 (1.16-1.38) | - | 1.25 (1.15-1.36) | 1.29 (0.86-1.95) |
| Free school meals eligible^b^ (ref=No) | 23,692 / 7,608 (32) | 1.00 | 1.00 | 1.00 | 1.00 | 1.00 |
| Persistent: at KS1 & KS2 (%) | 3,166 / 2,238 (71) | 4.49 (4.13-4.88) | 2.30 (2.07-2.55) | 2.28 (2.06-2.53) | 2.17 (1.96-2.41) | 1.66 (1.06-2.60) |
| At KS1 and not KS2 (%) | 1,609 / 985 (61) | 3.01 (2.70-3.35) | 1.80 (1.58-2.04) | 1.82 (1.61-2.06) | 1.77 (1.56-2.01) | 1.45 (0.77-2.70) |
| At KS2 and not KS1 (%) | 1,601 / 962 (60) | 2.87 (2.58-3.19) | 1.73 (1.52-1.96) | 1.75 (1.55-1.99) | 1.69 (1.49-1.92) | 1.59 (0.88-2.89) |
| Sex= male (%) | 15,584 / 6,800 (44) | 1.46 (1.39-1.53) | 1.11 (1.05-1.17) |  | 1.12 (1.05-1.18) |  |
| Townsend deprivation quintile at birth^b^ (ref=1 – least) | 4,968 / 1,133 (23) | 1.00 | 1.00 | 1.00 | 1.00 | 1.00 |
| 2 (%) | 5,518 / 1,747 (32) | 1.39 (1.26-1.52) | 1.23 (1.10-1.37) | 1.24 (1.11 - 1.38) | 1.24 (1.11-1.38) | 1.38 (0.61 - 3.12) |
| 3 (%) | 6,039 / 2,309 (38) | 1.76 (1.60-1.92) | 1.35 (1.22-1.51) | 1.37 (1.23 - 1.53) | 1.36 (1.22-1.51) | 1.30 (0.64 - 2.67) |
| 4 (%) | 6,230 / 2,775 (45) | 2.23 (2.04-2.44) | 1.54 (1.38-1.70) | 1.56 (1.40 - 1.73) | 1.54 (1.39-1.71) | 1.11 (0.56 - 2.20) |
| 5 - most (%) | 7, 531 / 4,044 (54) | 2.98 (2.72-3.25) | 1.69 (1.52-1.88) | 1.71 (1.54 - 1.90) | 1.69 (1.52-1.88) | 1.16 (0.62 - 2.19) |
| Congenital anomaly (ref=No) | 28,907 /11,368 (39) | 1.00 | 1.00 | 1.00 | 1.00 | 1.00 |
| minor (%) | 240 / 108 (45) | 1.21 (0.93-1.58) | 1.03 (0.75-1.43) | 1.00 (0.72 - 1.39) | 1.02 (0.74-1.41) | 0.60 (0.14 - 2.62) |
| major (%) | 1,292 / 588 (46) | 1.30 (1.16-1.47) | 0.94 (0.81-1.08) | 0.94 (0.81 - 1.08) | 0.93 (0.80-1.07) | 0.40 (0.20 - 0.80) |
| Maternal age at childbirth^b^ (ref=25-29 years) | 9,245 / 3,434 (37) | 1.00 | 1.00 | 1.00 | 1.00 | 1.00 |
| <25 (%) | 9,191 / 4,927 (54) | 1.78 (1.67-1.89) | 1.33 (1.23-1.43) | 1.32 (1.23 - 1.43) | 1.31 (1.22-1.42) | 1.03 (0.66 - 1.59) |
| 30-34 (%) | 8,242 / 2,520 (31) | 0.79 (0.74-0.84) | 0.85 (0.79-0.92) | 0.85 (0.79 - 0.92) | 0.85 (0.79-0.92) | 0.88 (0.50 - 1.55) |
| 35+ (%) | 3,740 / 1,173 (31) | 0.84 (0.77-0.92) | 0.89 (0.80-0.98) | 0.89 (0.80 - 0.98) | 0.89 (0.81-0.98) | 0.54 (0.26 - 1.10) |
| Academic season of birth (ref=Sep to Dec) | 8,170 / 2,957 (36) | 1.00 | 1.00 | 1.00 | 1.00 | 1.00 |
| Jan to Apr (%) | 10,690 / 4,235 (40) | 1.16 (1.09-1.24) | 1.06 (0.98-1.15) | 1.07 (0.99 - 1.16) | 1.07 (0.99-1.16) | 1.31 (0.83 - 2.08) |
| May to Aug (%) | 11,579 / 4,872 (42) | 1.28 (1.21-1.36) | 1.11 (1.03-1.21) | 1.12 (1.03 - 1.21) | 1.12 (1.03-1.21) | 1.42 (0.89 - 2.26) |
| Gestation at birth^c^ (ref=37+ weeks) | 26,425 / 10,283 (39) | 1.00 | 1.00 | 1.00 | 1.00 | 1.00 |
| 24-27 (%) | 70 / 37 (53) | 1.74 (1.07-2.82) | 1.20 (0.66-2.16) | 1.20 (0.66 - 2.17) | 1.22 (0.67-2.20) | - |
| 28-32 (%) | 382 / 197 (52) | 1.58 (1.28-1.95) | 1.08 (0.83-1.39) | 1.06 (0.82 - 1.38) | 1.07 (0.82-1.38) | 1.04* (0.30 - 3.64) |
| 33-36 (%) | 1,703 / 728 (43) | 1.18 (1.06-1.31) | 0.97 (0.85-1.10) | 0.97 (0.85 - 1.10) | 0.96 (0.84-1.10) | 1.30 (0.65 - 2.61) |
| Small for gestational age^c^ (<10^th^ centile) =yes (%) | 2,821 / 1,412 (50) | 1.56 (1.41-1.74) | 1.20 (1.04-1.39) | 1.19 (1.03 - 1.37) | 1.19 (1.03-1.37) | 0.94 (0.46 - 1.90) |
| Maternal smoking in first trimester (ref=No) | 3,418 / 1,126 (33) | 1.00 | 1.00 | 1.00 | 1.00 | 1.00 |
| Yes (%) | 1,235 / 706 (57) | 2.55 (2.40-2.72) | 1.55 (1.36-1.78) | 1.58 (1.38 - 1.82) | 1.55 (1.36-1.78) | 1.59 (0.98 - 2.57) |
| NA (%) | 25,786 / 10,232 (40) | - | - |  | - |  |
| SEN provision at KS2^b^ (ref=None) | 22,356 / 5,994 (27) | 1.00 | 1.00 | 1.00 | 1.00 | 1.00 |
| School action (%) | 4,409 / 3,194 (72) | 7.84 (7.27-8.46) | 5.03 (4.62-5.48) | 5.00 (4.59 - 5.45) | 5.01 (4.60-5.46) | 3.89 (2.26 - 6.69) |
| School action plus (%) | 2,524 / 1,974 (78) | 10.63 (9.58-11.79) | 6.03 (5.37-6.77) | 5.91 (5.26 - 6.64) | 5.93 (5.28-6.66) | 3.39 (1.89 - 6.10) |
| Statemented (%) | 887 / 755 (85) | 17.17 (14.10-20.89) | 11.99 (8.70-16.53) | 10.90 (7.90 - 15.03) | 10.91 (7.90-15.06) | 7.88 (2.52 - 24.66) |
| SEN provision at KS1^b^ (ref=None) | 22,510 / 6,596 (29) | 1.00 | 1.00 | 1.00 | 1.00 | 1.00 |
| School action (%) | 4,372 / 3,031 (69) | 5.54 (5.15-5.95) | 2.41 (2.21-2.63) | 2.43 (2.22 - 2.64) | 2.41 (2.21-2.62) | 2.12 (1.25 - 3.58) |
| School action plus (%) | 2,009 / 1,523 (76) | 7.71 (6.91-8.60) | 2.38 (2.09-2.72) | 2.38 (2.08 - 2.72) | 2.36 (2.07-2.70) | 4.03 (1.90 - 8.56) |
| Statemented (%) | 494 / 393 (80) | 9.96 (7.88-12.60) | 1.25 (0.84-1.86) | 1.18 (0.79 - 1.76) | 1.18 (0.79-1.77) | 2.21 (0.60 - 8.19) |
| Days absent in year take KS2^b^ (ref=None) | 3,542 / 1,185 (34) | 1.00 | 1.00 | 1.00 | 1.00 | 1.00 |
| 1 to 5 (%) | 7,120 / 2,121 (30) | 0.89 (0.81-0.97) | 0.96 (0.87-1.07) | 0.97 (0.87 - 1.07) | 0.97 (0.87-1.08) | 0.77 (0.41 - 1.45) |
| 6 to 10 (%) | 7,139 / 2,584 (36) | 1.16 (1.06-1.27) | 1.13 (1.02-1.25) | 1.14 (1.03 - 1.26) | 1.14 (1.03-1.26) | 1.20 (0.65 - 2.21) |
| 11 to 16 (%) | 5,722 / 2,365 (41) | 1.42 (1.29-1.55) | 1.25 (1.13-1.39) | 1.28 (1.15 - 1.42) | 1.26 (1.14-1.41) | 1.12 (0.60 - 2.12) |
| 17+ (%) | 6,653 / 3,662 (55) | 2.36 (2.16-2.58) | 1.67 (1.50-1.85) | 1.72 (1.55 - 1.90) | 1.68 (1.51-1.86) | 1.27 (0.70 - 2.29) |
| Number of schools to age 15 years (ref=1-2) | 15,860 / 5,606 (35) | 1.00 | 1.00 | 1.00 | 1.00 | 1.00 |
| 3 (%) | 9,676 / 3,941 (41) | 1.19 (1.12-1.26) | 1.06 (0.99-1.14) | 1.05 (0.98 - 1.13) | 1.05 (0.98-1.13) | 1.19 (0.77 - 1.84) |
| 4 (%) | 3,638 / 1,754 (48) | 1.46 (1.35-1.59) | 1.12 (1.02-1.24) | 1.12 (1.01 - 1.24) | 1.10 (1.00-1.22) | 1.11 (0.67 - 1.84) |
| 5 (%) | 974 / 560 (58) | 1.97 (1.71-2.28) | 1.23 (1.03-1.47) | 1.17 (0.98 - 1.39) | 1.15 (0.97-1.38) | 1.24 (0.67 - 2.27) |
| 6+ (%) | 291 / 203 (70) | 3.47 (2.66-4.51) | 2.02 (1.49-2.74) | 1.87 (1.38 - 2.54) | 1.79 (1.32-2.44) | 3.43 (1.32 - 8.91) |
| Year at age 16 years |  |  |  | 1.00 |  | 1.00 |
| 2014 | 7116 / 3109 (25.8) | 1.19 (1.11 - 1.26) | 1.27 (1.12 - 1.44) | 1.27 (1.11 - 1.45) | 1.27 (1.12 - 1.44) | 1.49 (0.88 - 2.49) |
| 2015 (ref) | 10881 / 4272 (39.3) | 1.00 | 1.00 | 1.00 | 1.00 | 1.00 |
| 2016 | 11098 / 4153 (37.4) | 0.90 (0.85 - 0.95) | 0.86 (0.80 - 0.92) | 0.86 (0.80 - 0.92) | 0.86 (0.80 - 0.92) | 0.91 (0.61 - 1.34) |
| 2017 | 1344 / 530 (39.4) | 0.97 (0.86 - 1.10) | 1.18 (1.02 - 1.37) | 1.20 (1.03 - 1.39) | 1.19 (1.02 - 1.38) | 1.78 (0.68 - 4.64) |

^a^ school year (reference year 2015), Social Care intervention and Adverse Childhood Experiences;^b^ <5% missing data; ^c^< 7% missing data.

**Supplementary Table 3 - Cox regression (Table 2 continued for confounders) for Social Care intervention between 12 to 15 years, exposure to Adverse Childhood Experiences to age 12 years and first all-cause emergency admission after age 15 years on 31^st^ March (Social Care Census date) and < 20 years outcome, N=30,439**

|  | **Proportion (%)** | **Unadjusted**  **HR (95% CI)** |  | **Multivariable: ACEs adjusted for other variables^a^**  **HR (95% CI)** | **Multivariable: Social Care adjusted for other variables^a^**  **HR (95% CI)** | **Multivariable: ACEs & Social Care adjusted for other variables^a^**  **HR (95% CI)** | **Social care data only**  **Multivariable: ACEs & Social Care adjusted for other variables^a^**  **HR (95% CI)** |
| --- | --- | --- | --- | --- | --- | --- | --- |
| **N** |  |  |  |  |  |  |  |
| Highest level of Social Care intervention age 12 to 15 years (ref=None) | 3836 (93.1) | 1.00 |  | - |  |  |  |
| Other children in need (%) | 178 (4.3) | 2.02 (1.74 - 2.35) |  | - | 1.67 (1.42 - 1.95) | 1.62 (1.38 - 1.90) |  |
| Child protection register (%) | 43 (1.0) | 2.07 (1.53 - 2.79) |  | - | 1.54 (1.14 - 2.10) | 1.51 (1.11 - 2.04) | 0.99 (0.70 - 1.41) |
| Children looked after: out-of-home care (%) | 65 (1.6) | 1.77 (1.38 - 2.26) |  | - | 1.37 (1.07 - 1.76) | 1.31 (1.01 - 1.68) | 0.78 (0.57 - 1.07) |
| **Ever a potential child adversity to age 11 years:** | | | | | | | |
| A victimisation hospital admission=yes (%) | 60 (1.5) | 1.61 (1.25 - 2.08) |  | 1.28 (0.99 - 1.66) | - | 1.23 (0.95 - 1.60) | 1.37 (0.84 - 2.23) |
| Household member with serious mental illness=yes (%) | 73 (1.8) | 1.38 (1.10 - 1.75) |  | 1.12 (0.88 - 1.41) | - | 1.10 (0.87 - 1.39) | 1.44 (0.85- 2.43) |
| A change to a single adult household=yes (%) | 1148 (27.9) | 1.25 (1.17 - 1.34) |  | 1.07 (1.00 - 1.15) | - | 1.07 (0.99 - 1.15) | 0.84 (0.65 - 1.09) |
| Death in the household child aged 1 to 11 years=yes (%) | 338 (8.2) | 1.18 (1.06 - 1.32) |  | 1.08 (0.96 - 1.21) |  | 1.07 (0.96 - 1.20) | 0.99 (0.68 - 1.44) |
| Household member with a common mental disorder |  | 1.00 |  |  | - |  |  |
| History to < 5 years=yes (%) | 1423 (34.5) | 1.22 (1.14 - 1.30) |  | 1.09 (1.01 - 1.17) | - | 1.09 (1.01 - 1.17) | 1.31 (1.01 - 1.68) |
| 5 to < 12 years=yes (%) | 1897 (46.0) | 1.22 (1.15 - 1.30) |  | 1.11 (1.04 - 1.19) | - | 1.11 (1.03 - 1.18) | 0.82 (0.64 - 1.07) |
| Household member with an alcohol problem |  | 1.00 |  |  | - |  |  |
| History to < 5 years=yes (%) | 445 (10.8) | 1.28 (1.16 - 1.42) |  | 1.03 (0.93 - 1.15) | - | 1.02 (0.91 - 1.13) | 1.19 (0.90 - 1.59) |
| 5 to < 12 years=yes (%) | 680 (16.5) | 1.27 (1.17 - 1.38) |  | 1.06 (0.97 - 1.16) | - | 1.05 (0.96 - 1.14) | 0.77 (0.58 - 1.02) |
| Free school meals eligible^b^ (ref=No) | 2906 (70.5) | 1.00 |  |  |  |  |  |
| Persistent: at KS1 & KS2 (%) | 600 (14.6) | 1.58 (1.45 - 1.73) |  | 1.22 (1.10 - 1.35) | 1.23 (1.11 - 1.36) | 1.17 (1.06 - 1.30) | 0.92 (0.67 - 1.26) |
| At KS1 and not KS2 (%) | 285 (6.9) | 1.46 (1.29 - 1.65) |  | 1.20 (1.05 - 1.36) | 1.23 (1.08 - 1.40) | 1.18 (1.04 - 1.35) | 1.23 (0.80 - 1.90) |
| At KS2 and not KS1 (%) | 247 (6) | 1.29 (1.13 - 1.46) |  | 1.05 (0.91 - 1.20) | 1.07 (0.94 - 1.23) | 1.03 (0.90 - 1.18) | 1.41 (0.96 - 2.06) |
| Sex= male (%) | 1767 (42.9) | 0.69 (0.65 - 0.73) |  | 0.67 (0.63 - 0.71) | 0.67 (0.63 - 0.71) | 0.67 (0.63 - 0.71) | 0.62 (0.48 - 0.80) |
| Townsend deprivation quintile at birth^b^  (ref=1 – least) | 541 (13.1) |  |  |  |  |  |  |
| 2 (%) | 696 (16.9) | 1.15 (1.03 - 1.29) |  | 1.10 (0.98 -1.23) | 1.10 (0.99 - 1.23) | 1.10 (0.99 - 1.23) | 0.62 (0.34 - 1.12) |
| 3 (%) | 795 (19.3) | 1.20 (1.08 - 1.34) |  | 1.07 (0.96 -1.20) | 1.08 (0.97 - 1.21) | 1.07 (0.96 - 1.20) | 0.77 (0.46 - 1.28) |
| 4 (%) | 924 (22.4) | 1.35 (1.22 - 1.50) |  | 1.15 (1.03 - 1.28) | 1.16 (1.04 - 1.29) | 1.15 (1.03 - 1.28) | 0.80 (0.49 - 1.30) |
| 5 - most (%) | 1152 (27.9) | 1.39 (1.26 - 1.54) |  | 1.08 (0.97 - 1.21) | 1.09 (0.98 - 1.21) | 1.08 (0.97 - 1.21) | 0.85 (0.54 - 1.34) |
| Congenital anomaly (ref=No) | 3849 (93.4) |  |  |  |  |  |  |
| minor (%) | 43 (1.0) | 1.36 (1.00 - 1.83) |  | 1.45 (1.07 - 1.96) | 1.43 (1.06 - 1.94) | 1.43 (1.06 - 1.94) | 2.07 (0.88 - 4.84) |
| major (%) | 230 (5.6) | 1.38 (1.21 - 1.57) |  | 1.39 (1.21 - 1.59) | 1.39 (1.21 - 1.59) | 1.38 (1.20 - 1.58) | 1.03 (0.64 - 1.67) |
| Maternal age at childbirth^b^ (ref=25-29 years) | 1228 (29.8) | 1.00 |  | 1.00 | 1.00 | 1.00 | 1.00 |
| 30-34 (%) | 972 (23.6) | 0.89 (0.82- 0.97) |  | 0.93 (0.85 - 1.01) | 0.92 (0.85 - 1.00) | 0.93 (0.85 - 1.01) | 1.00 (0.68 - 1.46) |
| 35+ (%) | 391 (9.5) | 0.79 (0.70 - 0.88) |  | 0.82 (0.73 - 0.92) | 0.82 (0.73 - 0.92) | 0.82 (0.73 - 0.92) | 1.12 (0.65 - 1.94) |
| <18 (%) | 186 (4.5) | 1.40 (1.20 - 1.64) |  | 1.16 (0.99 - 1.36) | 1.17 (1.00 - 1.37) | 1.15 (0.98 - 1.34) | 0.99 (0.61 - 1.61) |
| 18-24 (%) | 1340 (32.5) | 1.24 (1.15 - 1.34) |  | 1.12 (1.04 - 1.22) | 1.13 (1.05 - 1.23) | 1.12 (1.03 - 1.21) | 1.04 (0.77 - 1.41) |
| Gestation at birth^c^ (ref=37+ weeks) | 3545 (86.0) | 1.00 |  | 1.00 | 1.00 | 1.00 | 1.00 |
| 24 - <33 weeks (%) | 69 (1.7) | 1.13 (0.89 - 1.43) |  | 1.02 (0.80 - 1.30) | 1.02 (0.81 - 1.30) | 1.03 (0.81 - 1.30) | 1.39 (0.72 - 2.70) |
| 33 - <37 (%) | 260 (6.3) | 1.13 (0.99 - 1.28) |  | 1.10 (0.96 - 1.25) | 1.10 (0.97 - 1.25) | 1.09 (0.96 - 1.25) | 0.82 (0.52 - 1.30) |
| Small for gestational age^c^ (<10^th^ centile) =yes (%) | 429 (10.4) | 1.13 (0.98 - 1.29) |  | 1.02 (0.89 - 1.17) | 1.01 (0.88 - 1.16) | 1.02 (0.88 - 1.16) | 0.92 (0.63 - 1.35) |
| Maternal smoking in first trimester (ref=No) | 415 (10.1) | 1.00 |  | 1.00 | 1.00 | 1.00 | 1.00 |
| Yes (%) | 203 (4.9) | 1.37 (1.24 - 1.50) |  | 1.16 (1.03 - 1.31) | 1.18 (1.04 - 1.33) | 1.16 (1.03 - 1.31) | 1.07 (0.76 - 1.52) |
| NA (%) | 3504 (85.0) |  |  |  |  |  |  |
| SEN provision at KS2^b^ (ref=None) | 2857 (69.3) | 1.00 |  | 1.00 | 1.00 | 1.00 | 1.00 |
| School action (%) | 700 (17.0) | 1.25 (1.15 - 1.35) |  | 1.16 (1.06 - 1.27) | 1.15 (1.05 - 1.27) | 1.15 (1.05 - 1.26) | 1.01 (0.71 - 1.44) |
| School action plus (%) | 395 (9.6) | 1.23 (1.10 - 1.36) |  | 1.17 (1.03 - 1.31) | 1.14 (1.01 - 1.29) | 1.14 (1.01 - 1.29) | 0.80 (0.54 - 1.18) |
| Statemented (%) | 129 (3.1) | 1.12 (0.93 - 1.33) |  | 1.09 (0.83 - 1.42) | 1.00 (0.76 - 1.31) | 1.00 (0.76 - 1.31) | 0.72 (0.39 - 1.32) |
| SEN provision at KS1^b^ (ref=None) | 2920 (70.8) | 1.00 |  | 1.00 | 1.00 | 1.00 | 1.00 |
| School action (%) | 659 (16.0) | 1.18 (1.08 - 1.28) |  | 1.04 (0.95 - 1.15) | 1.05 (0.95 - 1.15) | 1.04 (0.94 - 1.14) | 1.09 (0.78 - 1.53) |
| School action plus (%) | 302 (7.3) | 1.16 (1.03 - 1.30) |  | 1.02 (0.89 - 1.17) | 1.02 (0.89 - 1.16) | 1.01 (0.88 - 1.16) | 1.26 (0.84 - 1.89) |
| Statemented (%) | 69 (1.7) | 1.10 (0.87 - 1.38) |  | 0.97 (0.69 - 1.37) | 0.93 (0.66 - 1.32) | 0.93 (0.66 - 1.32) | 1.10 (0.54 - 2.27) |
| Year at age 16 years |  |  |  |  |  |  |  |
| 2014 | 1079 (26.2) | 0.99 (0.92 - 1.07) |  | 0.99 (0.91 - 1.08) | 0.99 (0.91 - 1.07) | 0.99 (0.91 - 1.08) | 0.98 (0.71 - 1.35) |
| 2015 (ref) | 1626 (39.4) | 1.00 |  | 1.00 | 1.00 | 1.00 | 1.00 |
| 2016 | 1289 (31.3) | 0.94 (0.88 - 1.02) |  | 0.94 (0.88 - 1.02) | 0.95 (0.88 - 1.02) | 0.95 (0.88 - 1.02) | 1.00 (0.75 - 1.33) |
| 2017 | 128 (3.1) | 0.91 (0.76 - 1.09) |  | 0.91 (0.76 - 1.09) | 0.92 (0.77 - 1.10) | 0.91 (0.76 - 1.09) | 1.49 (0.85 - 2.60) |

^a^ school year (reference year 2015), Social Care intervention and Adverse Childhood Experiences;^b^ <5% missing data; ^c^< 7% missing data.

**Supplementary Table 4 - Cox regression (Table 3 continued for confounders) for Social Care intervention between 12 to 15 years, exposure to Adverse Childhood Experiences to age 12 years and first injury or external cause emergency admission after age 15 years on 31^st^ March (Social Care Census date) and < 20 years outcome, N=30,439**

|  | **Proportion (%)** | **Unadjusted**  **HR (95% CI)** | **Multivariable: ACEs adjusted for other variables^a^**  **HR (95% CI)** | **Multivariable: Social Care adjusted for other variables^a^**  **HR (95% CI)** | **Multivariable: ACEs & Social Care adjusted for other variables^a^**  **HR (95% CI)** | **Multivariable: ACEs & Social Care adjusted for other variables^a^**  **HR (95% CI)**  **(Interactions)** | **Social care data only**  **Multivariable: ACEs & Social Care adjusted for other variables^a^**  **HR (95% CI)** |
| --- | --- | --- | --- | --- | --- | --- | --- |
| **N** |  |  |  |  |  |  |  |
| Highest level of Social Care intervention age 12 to 15 years (ref=None) | 1071 (90.5) | 1.00 | - | 1.00 | 1.00 | 1.00 | - |
| Other children in need (CIN) / Child protection register | 81 (7.0) | 2.56 (2.04 - 3.21) | - | 2.19 (1.72 - 2.78) | 2.09 (1.64 - 2.66) | 2.07 (1.50 - 2.85) | 1.00 |
| (CPR) (%) | 31 (2.6) | 2.96 (2.07 - 4.23) | - | 2.53 (1.75 - 3.66) | 2.33 (1.60 - 3.39) | 1.41 (0.77 - 2.60) | 0.71 (0.35 - 1.43) |
| **Ever a potential child adversity to age 11 years:** | | | | | | |  |
| A victimisation hospital admission=yes (%) | 18 (1.5) | 1.64 (1.03 - 2.60) | 1.26 (0.79 – 2.02) | - | 1.10 (0.69 - 1.78) | 1.13 (0.70 - 1.81) | 0.84 (0.33 - 2.12) |
| Household member with serious mental illness=yes (%) | 20 (1.7) | 1.30 (0.83 - 2.02) | 1.02 (0.65 - 1.60) | - | 0.98 (0.63 - 1.53) | 0.98 (0.63 - 1.53) | 0.99 (0.39 - 2.51) |
| A change to a single adult household=yes (%) | 354 (29.9) | 1.37 (1.21 - 1.55) | 1.14 (1.00 - 1.30) | - | 1.13 (0.99 - 1.29) | 1.13 (0.99 - 1.29) | 0.82 (0.55 - 1.23) |
| Death in the household child aged 1 to 11 years=yes (%) | 115 (9.7) | 1.42 (1.17 - 1.72) | 1.24 (1.02 - 1.51) |  | 1.23 (1.01 - 1.49) | 1.23 (1.01 - 1.49) | 0.82 (0.55 - 1.23) |
| Household member with a common mental disorder |  | 1.00 | 1.00 | - | 1.00 | 1.00 | 1.00 |
| History to < 5 years=yes (%) | 4.08 (34.5) | 1.20 (1.07 - 1.36) | 1.05 (0.92 - 1.20) | - | 1.05 (0.92 - 1.19) | 1.02 (0.88 - 1.17) | 1.05 (0.65 - 1.68) |
| Interaction: History to < 5 years X Other CIN/CPR=yes(%) | - | - | - | - | - | 1.02 (0.65 - 1.62) | - |
| Interaction: History to < 5 years= X CLA=yes(%) | - | - | - | - | - | 2.52 (1.19 - 5.34) | 2.41 (1.00 - 5.79) |
| 5 to < 12 years=yes (%) | 553 (46.7) | 1.25 (1.11 - 1.40) | 1.12 (0.98 - 1.27) | - | 1.11 (0.98 - 1.26) | 1.12 (0.98 - 1.26) | 1.11 (0.73 - 1.69) |
| Household member with an alcohol problem |  | 1.00 | 1.00 | - | 1.00 | 1.00 | 1.00 |
| History to < 5 years=yes (%) | 145 (12.3) | 1.47 (1.23 - 1.74) | 1.47 (1.09 - 1.98) | - | 1.06 (0.88 - 1.28) | 1.41 (1.05 - 1.90) | 1.16 (0.57 - 2.35) |
| Interaction: History to < 5 years X Time to outcome (per year) |  | 0.98 (0.95 – 0.996) | 0.98 (0.95 – 1.00) | 1.00 (0.99 – 1.01) | - | 0.98 (0.96 - 1.00) | 1.00 (0.95 - 1.05) |
| 5 to < 12 years=yes (%) | 227 (19.2) | 1.52 (1.32 - 1.76) | 1.22 (1.05 - 1.43) | - | 1.18 (1.01 - 1.38) | 1.18 (1.01 - 1.38) | 0.75 (0.48 - 1.17) |
| Free school meals eligible^b^ (ref=No) | 812 (68.6) | 1.00 | 1.00 | 1.00 | 1.00 | 1.00 | 1.00 |
| Persistent: at KS1 & KS2 (%) | 188 (15.9) | 1.75 (1.48 - 2.05) | 1.32 (1.08 - 1.61) | 1.32 (1.08 - 1.61) | 1.22 (1.00 - 1.49) | 1.22 (1.00 - 1.49) | 1.08 (0.65 - 1.78) |
| At KS1 and not KS2 (%) | 84 (7.1) | 1.55 (1.24 - 1.94) | 1.25 (0.99 - 1.58) | 1.27 (1.01 - 1.61) | 1.20 (0.95 - 1.52) | 1.21 (0.96 - 1.53) | 1.16 (0.57 - 2.35) |
| At KS2 and not KS1 (%) | 72 (6.1) | 1.33 (1.04 - 1.70) | 1.05 (0.81 - 1.36) | 1.07 (0.83 - 1.38) | 1.01 (0.78 - 1.30) | 1.01 (0.78 - 1.31) | 1.22 (0.64 - 2.32) |
| Sex= male (%) | 640 (54.1) | 1.11 (0.99 - 1.25) | 1.11 (0.98 - 1.24) | 1.12 (1.00 - 1.26) | 1.12 (1.00 - 1.26) | 1.12 (1.00 - 1.26) | 0.89 (0.60 - 1.32) |
| Townsend deprivation quintile at birth^b^ (ref=1 – least) | 147 (12.4) | 1.00 | 1.00 | 1.00 | 1.00 | 1.00 | 1.00 |
| 2 (%) | 209 (17.7) | 1.26 (1.02 - 1.56) | 1.19 (0.96 - 1.47) | 1.20 (0.97 - 1.48) | 1.20 (0.97 - 1.48) | 1.19 (0.97 - 1.48) | 0.40 (0.13 - 1.21) |
| 3 (%) | ~240 (~20.0) | 1.32 (1.08 - 1.62) | 1.15 (0.94 - 1.42) | 1.17 (0.95 - 1.44) | 1.16 (0.94 - 1.43) | 1.16 (0.94 - 1.43) | 0.98 (0.44 - 2.17) |
| 4 (%) | 245 (20.7) | 1.30 (1.06 - 1.59) | 1.06 (0.86 - 1.31) | 1.08 (0.87 - 1.33) | 1.06 (0.86 - 1.31) | 1.06 (0.86 - 1.31) | 0.80 (0.37 - 1.74) |
| 5 - most (%) | 339 (28.7) | 1.48 (1.22 - 1.80) | 1.09 (0.89 - 1.34) | 1.10 (0.90 - 1.35) | 1.09 (0.88 - 1.33) | 1.09 (0.89 - 1.34) | 0.86 (0.41 - 1.79) |
| Congenital anomaly (ref=No) | 1119 (94.6) | 1.00 | 1.00 | 1.00 | 1.00 | 1.00 | 1.00 |
| minor (%) | 10 (0.8) | 1.05 (0.56 - 1.96) | 1.04 (0.55 - 1.93) | 0.99 (0.53 - 1.86) | 1.01 (0.54 - 1.88) | 1.00 (0.54 - 1.87) | 0.75 (0.10 - 5.55) |
| major (%) | 54 (4.6) | 1.08 (0.82 - 1.42) | 1.10 (0.83 - 1.45) | 1.11 (0.84 - 1.47) | 1.10 (0.83 - 1.45) | 1.10 (0.83 - 1.45) | 0.97 (0.43 - 2.19) |
| Maternal age at childbirth^b^ (ref=25-29 years) | 346 (29.2) | 1.00 | 1.00 | 1.00 | 1.00 | 1.00 | 1.00 |
| 30-34 (%) | 265 (22.4) | 0.87 (0.74 - 1.02) | 0.91 (0.78 - 1.07) | 0.90 (0.77 - 1.06) | 0.91 (0.78 - 1.07) | 0.91 (0.78 - 1.07) | 0.98 (0.54 - 1.77) |
| 35+ (%) | ~112 (9.8) | 0.84 (0.68 - 1.04) | 0.89 (0.72 - 1.10) | 0.88 (0.71 - 1.09) | 0.89 (0.72 - 1.10) | 0.90 (0.73 - 1.11) | 0.93 (0.38 - 2.29) |
| <18 (%) | 56 (4.7) | 1.48 (1.12 - 1.97) | 1.16 (0.87 - 1.56) | 1.20 (0.90 - 1.60) | 1.14 (0.86 - 1.53) | 1.14 (0.85 - 1.53) | 0.93 (0.44 - 1.96) |
| 18-24 (%) | 399 (33.7) | 1.30 1.13 1.51 | 1.15 0.99 1.33 | 1.16 1.00 1.34 | 1.13 0.97 1.31 | 1.13 0.97 1.31 | 0.85 0.53 1.37 |
| Gestation at birth^c^ (ref=37+ weeks) | 1022 (86.4) | 1.00 | 1.00 | 1.00 | 1.00 | 1.00 | 1.00 |
| 24 - <33 weeks (%) | 15 (1.3) | 0.84 (0.50 - 1.40) | 0.79 (0.47 - 1.32) | 0.79 (0.47 - 1.32) | 0.80 (0.48 - 1.33) | 0.79 (0.47 - 1.32) | 1.41 (0.48 - 4.11) |
| 33 - <37 (%) | 60 (5.1) | 0.92 (0.72 - 1.18) | 0.88 (0.69 - 1.14) | 0.88 (0.69 - 1.14) | 0.88 (0.68 - 1.13) | 0.89 (0.69 - 1.14) | 0.79 (0.36 - 1.71) |
| Small for gestational age^c^ (<10^th^ centile) =yes (%) | 107 (9.0) | 1.07 (0.86 - 1.32) | 0.97 (0.78 - 1.20) | 0.94 (0.76 - 1.17) | 0.94 (0.76 - 1.17) | 0.95 (0.76 - 1.17) | 0.84 (0.41 - 1.71) |
| Maternal smoking in first trimester (ref=No) | 122 (10.3) | 1.00 | 1.00 | 1.00 | 1.00 | 1.00 | 1.00 |
| Yes (%) | 71 (6.0) | 1.47 (1.25 - 1.74) | 1.21 (0.99 - 1.48) | 1.25 (1.03 - 1.51) | 1.21 (1.00 - 1.46) | 1.21 (1.00 - 1.46) | 1.14 (0.68 - 1.92) |
| NA (%) | 990 (7.3) |  |  |  |  |  |  |
| SEN provision at KS2^b^ (ref=None) | 807 (68.2) | 1.00 | 1.00 | 1.00 | 1.00 | 1.00 | 1.00 |
| School action (%) | 210 (17.8) | 1.32 (1.13 - 1.53) | 1.16 (0.98 - 1.36) | 1.14 (0.96 - 1.35) | 1.14 (0.96 - 1.34) | 1.14 (0.96 - 1.35) | 1.29 (0.75 - 2.23) |
| School action plus (%) | 121 (10.2) | 1.33 (1.10 - 1.61) | 1.16 (0.93 - 1.44) | 1.11 (0.89 - 1.38) | 1.10 (0.89 - 1.37) | 1.10 (0.89 - 1.36) | 0.86 (0.46 - 1.60) |
| Statemented (%) | 32 (2.7) | 0.98 (0.69 - 1.40) | 0.91 (0.53 - 1.56) | 0.77 (0.45 - 1.32) | 0.77 (0.45 - 1.33) | 0.78 (0.45 - 1.34) | 0.72 (0.27 - 1.94) |
| SEN provision at KS1^b^ (ref=None) | 831 (70.2) | 1.00 | 1.00 | 1.00 | 1.00 | 1.00 | 1.00 |
| School action (%) | 199 (16.8) | 1.25 (1.07 - 1.46) | 1.01 (0.85 - 1.20) | 1.00 (0.84 - 1.20) | 0.99 (0.83 - 1.18) | 1.00 (0.84 - 1.19) | 0.93 (0.53 - 1.61) |
| School action plus (%) | 86 (7.3) | 1.15 (0.92 - 1.43) | 0.91 (0.71 - 1.17) | 0.88 (0.69 - 1.14) | 0.88 (0.68 - 1.13) | 0.88 (0.68 - 1.13) | 1.03 (0.55 - 1.94) |
| Statemented (%) | 18 (1.5) | 0.95 (0.60 - 1.51) | 0.92 (0.46 - 1.82) | 0.84 (0.43 - 1.68) | 0.85 (0.43 - 1.69) | 0.85 (0.43 - 1.69) | 0.83 (0.25 - 2.69) |
| Year at age 16 years |  |  |  |  |  |  |  |
| 2014 | 293 (24.8) | 0.92 (0.80 - 1.07) | 0.92 (0.79 - 1.08) | 0.93 (0.80 - 1.08) | 0.93 (0.80 - 1.08) | 0.93 (0.80 - 1.08) | 1.29 (0.78 - 2.14) |
| 2015 (ref) | 472 (39.9) | 1.00 | 1.00 | 1.00 | 1.00 | 1.00 | 1.00 |
| 2016 | 381 (32.2) | 0.96 (0.84 - 1.10) | 0.96 (0.83 - 1.10) | 0.96 (0.84 - 1.11) | 0.96 (0.84 - 1.10) | 0.96 (0.84 - 1.10) | 1.32 (0.84 - 2.08) |
| 2017 | 37 (3.1) | 0.89 (0.63 - 1.24) | 0.87 (0.62 - 1.22) | 0.89 (0.64 - 1.25) | 0.88 (0.63 - 1.23) | 0.88 (0.63 - 1.23) | 1.10 (0.38 - 3.17) |

^a^ school year (reference year 2015), Social Care intervention and Adverse Childhood Experiences;^b^ <5% missing data; ^c^< 7% missing data; ~ denotes masked data.

**Supplementary Table 5: Sensitivity analysis for variables potentially on the pathway between ACEs, social care intervention and not attaining KS4 Level 2 (inclusive) in education**

|  | **Multivariable: ACEs & Social Care adjusted for other variables^a^ but not absence from school in KS2 year**  **cOR (95% CI)** | **Multivariable: ACEs & Social Care adjusted for other variables^a^ but not absence from school in KS2 year or number of schools attended**  **cOR (95% CI)** | **Multivariable: ACEs & Social Care adjusted for other variables^a^ but not absence from school in KS2 year or number of schools attended or SEN KS1 or 2**  **cOR (95% CI)** |
| --- | --- | --- | --- |
| **N** | 30,439 | 30,439 | 30,439 |
| Highest level of Social Care intervention age 12 to 15 years (ref=None) | 1.00 | 1.00 | 1.00 |
| Other children in need (%) | 2.53 (2.02-3.17) | 2.61 (2.08-3.27) | 3.98 (3.24-4.89) |
| Child protection register (%) | 4.26 (2.58-7.04) | 4.47 (2.71-7.37) | 4.95 (3.10-7.90) |
| Children looked after: out-of-home care (%) | 1.66 (1.17-2.33) | 1.77 (1.26-2.50) | 3.11 (2.27-4.26) |
| Ever a potential child adversity to age 11 years: |  |  |  |
| A victimisation hospital admission=yes (%) | 1.16 (0.85-.1.58) | 1.17 (0.86-1.60) | 1.46 (1.10-1.93) |
| Household member with serious mental illness=yes (%) | 0.81 (0.62-1.05) | 0.81 (0.63-1.06) | 0.89 (0.70-1.13) |
| A change to a single adult household=yes (%) | 0.99 (0.92-1.06) | 1.00 (0.93-1.07) | 1.00 (0.94-1.07) |
| Death in the household child age 1 to 11 years=yes (%) | 1.04 (0.93-1.16) | 1.04 (0.93-1.17) | 1.08 (0.98-1.19) |
| Household member with a common mental disorder |  |  |  |
| History to < 5 years=yes (%) | 1.04 (0.97-1.11) | 1.04 (0.97-1.11) | 1.06 (0.99-1.12) |
| 5 to < 12 years=yes (%) | 1.17 (1.10-1.25) | 1.18 (1.10-1.25) | 1.20 (1.13-1.27) |
| Household member with an alcohol problem |  |  |  |
| History to < 5 years=yes (%) | 1.07 (0.96-1.19) | 1.07 (0.96-1.19) | 1.10 (0.99-1.21) |
| 5 to < 12 years=yes (%) | 1.27 (1.16-1.38) | 1.28 (1.17-1.39) | 1.26 (1.16-1.37) |
| Free school meals eligible^b^ (ref=No) | 1.00 | 1.00 | 1.00 |
| Persistent: at KS1 & KS2 (%) | 2.27 (2.05-2.52) | 2.28 (2.06-2.53) | 2.66 (2.42-2.93) |
| At KS1 and not KS2 (%) | 1.82 (1.60-2.06) | 1.83 (1.61-2.08) | 2.01 (1.77-2.28) |
| At KS2 and not KS1 (%) | 1.75 (1.54-1.99) | 1.77 (1.56-2.01) | 1.89 (1.69-2.12) |
| Number of schools attended (ref=1 to 2) | 1.00 | 1.00 | 1.00 |
| 6+ (%) | 1.86 (1.37-2.54) | - | - |

^a^ school year (reference year 2015), sex, gestational age at birth, small for gestational age (<10^th^ centile), academic season of birth, congenital anomaly, maternal age at childbirth, maternal smoking in first trimester, deprivation quintile at birth, Special Educational Needs provision (SEN) at KS1, SEN at KS2, Free School Meals eligible at KS1 or KS2, number of schools attended, number of days absent in year take KS2; ^b^ <5% missing data
